# Supplementary figures and images for: L-Arginine reverses maternal and pre-pubertal codeine exposure-induced sexual dysfunction via upregulation of androgen receptor gene and NO/cGMP signaling
Source: PLoS One. 2022 Sep 13;17(9):e0274411. doi: 10.1371/journal.pone.0274411 (PMC9469994; doi:10.1371/journal.pone.0274411)

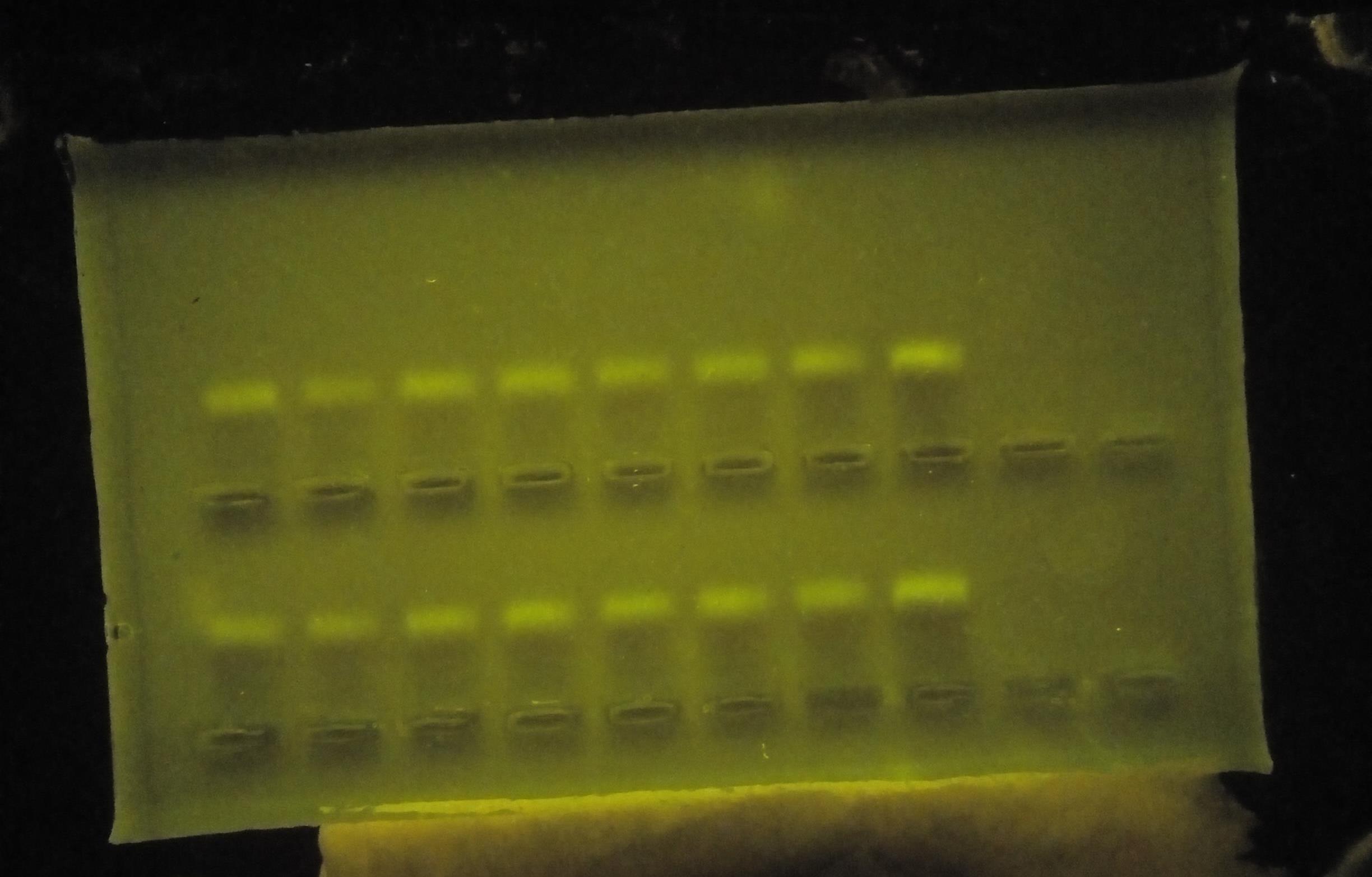


mRNA AR gel


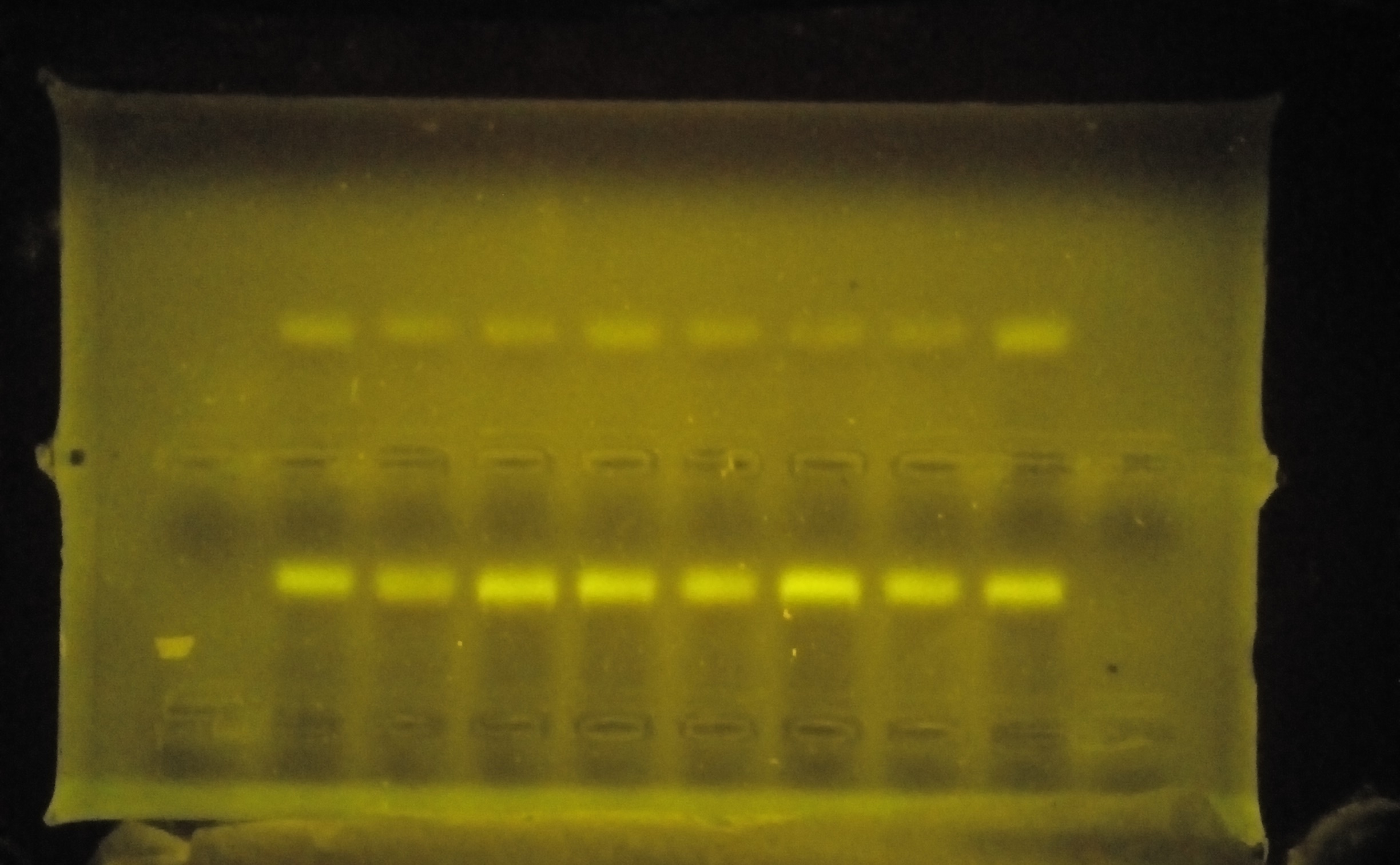


mRNA GAPDH

Supplement: S1 Raw images — (DOC) [file pone.0274411.s001.doc]
