# Supplementary material for: L-Arginine reverses maternal and pre-pubertal codeine exposure-induced sexual dysfunction via upregulation of androgen receptor gene and NO/cGMP signaling
Source: PLoS One. 2022 Sep 13;17(9):e0274411. doi: 10.1371/journal.pone.0274411 (PMC9469994; doi:10.1371/journal.pone.0274411)
Supplement: Attachment — Submitted filename: Comments.docx [file pone.0274411.s002.docx]

Comments：

PONE-D-22-06170: L-Arginine Reverses Maternal and Pre-pubertal Codeine Exposure-Induced Sexual Dysfunction via Upregulation of Androgen Receptor Gene and NO/cGMP Signaling.

The study evaluated the effect of maternal codeine exposure and prepubertal codeine and arginine treatments on F1 male sexual function and fertility indices, as well as the outcome of F2 progenies. In addition, the epigenetic programming mechanism was also explored, which is related to decreased dopamine analysis, decreased testosterone level, and inhibition of local GR expression in testis.In addition, when the male offspring of F1 generation were mated to F2 generation, pathological changes such as shortened fetal body length, weight loss and reduced number of live fetuses were observed.And these changes were reversed when given arginine.This study is helpful to guide the healthy life of male and to guide eugenics.In my opinion, the manuscript is innovative to a certain extent and needs to be revised accordingly. Specific opinions are as follows:

1、 It is important that the authors write this discussion without discussing the reality of codeine exposure during pregnancy or adolescence, the basis for modeling and the corresponding timing and duration of administration are also very important.
2、 When explaining the mechanism of pathological changes, the author uses AR as the research target, so what is the specific signal pathway that AR participates in this project? The author did not carry out corresponding detection, should not fill?
3、 AR is expressed in a variety of testicular cells, such as Sertoli cells, Leydig cells and epithelial cells. What kind of cells does codeine mainly affect AR? Are you considering further validation in cells?
4、 The result part of this paper is too monotonous, so the result part of the manuscript should be enriched, such as adding some morphological changes of testis, sperm quality and other related indicators;
5、 Why does this paper focus on dopamine? Is it due to the biological effects of codeine? The author needs to describe the corresponding reasons for selection in the discussion.

6、There are many irregularities in writing in the manuscript:

（1） The notation is not standardized, and no specific explanation is given for each small figure
（2） For the reference section, a unified format is required
（3） In the WB results, the sensation is not produced on the same experiment. It should be replaced.

7、There are many grammatical errors in the full text, which make it difficult to read the manuscript. Please check it.
